# Prospecting for efflux pump and repressor genes in rivers of the Amazon region of Brazil

**DOI:** 10.1007/s11033-026-12979-1

**Published:** 2026-10-23

**Authors:** Valéria Alves Chaves, David Tavares Martins, Carlos Willian Dias Dantas, Savia Moreira da Silva, Juliana Araripe Gomes da Silva, Ana Lídia Queiroz Cavalcante, Daralyns Borges Macedo, Ana Luzia Leite Fernandes, Sandeep Tiwari, Rommel Thiago Jucá Ramos, Oscar Victor Cardenas-Alegria

**Affiliations:** 1 Institute of Socioenvironmental and Water Resources, Federal Rural University of the Amazon, Belém, Brazil; 2 Institute of Biological Sciences, Federal University of Pará, Belém, Brazil; 3 Institute of Biological Sciences, Federal University of Minas Gerais, Belo Horizonte, 31275-013 Brazil; 4 Laboratory of Simulation and Computational Biology, Pará Center for High Performance Distributed Computing (CCAD), Federal University of Pará UFPA, Belém, Brazil; 5 Laboratory of Genetics and Conservation, Institute of Coastal Studies, Federal University of Pará (UFPA), Bragança, Brazil; 6 Institute of Biology (Microbiology) & Institute of Health Sciences, Federal Universityof Bahia (UFBA), Salvador, Brazil

**Keywords:** Efflux pumps, Repressors, River water, Multi-drug Resistance bacteria, Antibiotic gene resistance

## Abstract

**Background:**

The spread of multidrug-resistant bacteria in water bodies poses a significant public health concern. Efflux pumps represent critical mechanisms of antimicrobial resistance, regulated by genetic repressors that aid bacterial adaptation to environmental stress. This study profiles the diversity of efflux pump genes and genetic repressors in the surface waters of the Cereja (Bragança) and Uraim (Paragominas) rivers in the Amazon region of Brazil.

**Methods and Results:**

Surface water samples were collected along a gradient of anthropogenic impact, and environmental DNA was sequenced on the Illumina platform. The resulting sequencing data were subsequently subjected to metagenomic analysis, which identified a higher abundance of efflux pump genes in the Cereja River compared to the Uraim River, dominated by the RND and SMR families. Additionally, two-component system (TCS) repressor genes, particularly *cbrB*, were successfully recovered and modeled using Swiss-Model homology modeling.

**Conclusions:**

The higher coverage of the *cbrB* repressor gene in anthropogenically impacted sites underscores its regulatory role in bacterial stress responses, suggesting its potential application as a novel biomarker of environmental pollution and antibiotic resistance spread in aquatic ecosystems.

**Supplementary Information:**

The online version contains supplementary material available at https://doi.org/10.1007/s11033-026-12979-1.

## Introduction

The excessive use of antibiotics has facilitated the selection of resistant bacteria through different mechanisms, leading to the emergence of bacterial strains capable of resisting multiple antibiotics, known as multidrug-resistant bacteria (MDR) [1]. These bacteria are more prevalent in highly anthropogenic environments due to the increased presence of various chemical compounds, such as antibiotics, biocides, hydrocarbons, and heavy metals [2]. This makes it possible for cross-resistance to occur — when a single mechanism provides resistance to more than one agent from the same family or to different biocides [3].

Efflux pumps (EPs) act as resistance mechanisms and play an important role in resistance to antibiotics and other molecules, which may be specific, such as resistance to colistin [4]. These molecules catalyze the active efflux of various compounds, including antibiotics, organochlorine compounds, and metals, which are stressful substances for microorganisms [2]. EP genes can be embedded within mobile elements, such as plasmids or insertion elements, which ensures their dissemination and persistence in different bacterial communities [5]. Thus, EPs are involved in virulence and biofilm formation, acting in the transport of extracellular polymeric substances (EPSs) and/or quorum sensing (QS) molecules to assist in the formation of the biofilm matrix; they are involved in the indirect regulation of genes related to biofilm formation; they can expel harmful molecules, such as antibiotics and metabolic intermediates that could potentially damage or inhibit biofilm growth; and finally, EPs may influence aggregation by promoting or preventing adhesion to surfaces and other cells [6].

The regulatory mechanism of efflux pumps (EPs) can differ between species and even within a species, depending on the cellular physiological state. This regulation is complex and highly interconnected, such that the inhibition or deletion of one efflux pump may result in the expression of others [7]. In this context, two main pathways have been described in terms of the genetic regulation of efflux pumps: (i) regulation mediated by transcriptional activators or repressors with DNA-binding motifs, and (ii) activation of two-component systems (TCS), which regulate gene expression in different ways [7].

On the other hand, few studies are being developed in the Amazon region, which, unfortunately, remains underserved mainly by healthcare systems. In Brazil, this ecosystem — comprising the northern region of the country — is constantly changing due to various economic activities, which favor the emergence of diseases that affect both humans and animals [8]. In this region, the municipality of Bragança stands out as one of the oldest cities, with a significant contribution to the local economy through commerce and services [9]. The city has good coverage in terms of water availability for residents living in private households, with 55.22% having an exclusive bathroom and 73.73% having access to piped water from a public network, wells, springs, or reservoirs supplied by rainwater or water trucks [10]. In addition, the city is crossed by a river which, in contrast, receives inputs of various pollutants from domestic, commercial, and hospital sewage directly from illegal discharge sources — a consequence of a common issue in the Amazon region: the lack of basic sanitation and sewage systems [11].

The municipality of Paragominas stands out in the region as the largest producer of grains in Pará, particularly corn and soybeans. It also has intense mining activity, mainly of bauxite and, to a lesser extent, aluminum compounds, kaolin, and silver [12, 13]. The Uraim River basin is located in a region with various activities that pose environmental risks to both the qualitative and quantitative aspects of its waters. This river basin has undergone considerable deforestation of its native vegetation [14], leading to frequent flooding problems, which are further influenced by climate change [13].

In recent years, studies in the Amazon region have become increasingly frequent due to the importance of its biodiversity and also because of the changes the ecosystem has been undergoing. Consequently, these accelerated changes may be resulting in a potential source of microorganisms with new antibiotic resistance traits involving different genetic components, such as efflux pumps and repressors. This study provides a better understanding of the ability of bacteria to adapt to ecosystem stress. In this context, the purpose of this work is to genetically characterize efflux pumps and repressors in rivers with varying levels of pollutants, in order to assess the impact of this contamination on the diversity of these mechanisms and the potential emergence of public health threats.

## Methodology

### Samples

The samples were obtained from the Uraim and Cereja rivers, which flow through the municipalities of Paragominas and Bragança, respectively. Bragança, one of the oldest cities in the Amazon, boasts a rich historical and architectural heritage and is home to over 100,000 inhabitants [15]. The waters of the Cereja River are influenced by the Atlantic Ocean, with an acidic pH, and high concentrations of fecal coliforms. This river is 4 km long [16]. The municipality of Paragominas, located in the southeastern mesoregion of Pará, 320 km from the city of Belém, has an area of 19,330 square kilometers (1.5% of the surface of Pará). In this territory, various economic activities of the Amazon are carried out, including small, medium, and large scale agriculture, livestock farming, timber production, charcoal production, and bauxite mining. Reforestation is a recent activity and is currently in an expansion phase in the municipality [12].

Five sampling points were selected based on varying levels of anthropogenic impact: P1, P2, P3, P4, and P5. Point P1 was located in an untouched, pristine site, while the final point (P5) was located downstream after passing through the city. The remaining points were intermediate between these two extremes. The samples were collected at a depth of 20 cm, with an independent 5 L water sample collected at each point in triplicate (biological replicates), using a Van Dorn bottle. They were preserved on ice and immediately transported to the laboratory for analysis. Upon arrival, the water samples were filtered using a negative pressure filtration system, assisted by a vacuum pump and 47 mm membranes with a 0.22 μm porosity. These membranes were preserved in 50mL Falcon tubes containing TSE buffers (Tris HCl 10mM, pH = 8, Sodium Chloride 100mM, EDTA 1mM, pH = 8) and stored at−20° C.

Physical-chemical component measurements were conducted in situ using a multi parameter water analyzer (HI 9829, HANNA), considering the parameters of pH. The sampling points were chosen following a bioprospecting approach, with considera tion given to the gradient of anthropogenic impact and accessibility for sample collection. These points were situated at the outset of the pristine site (PP1), specifically at co ordinates 3° 3’ 28.512” S, 47° 25’ 2.172” W. Following the initial point, the subsequent sampling locations were designated as PP2 (3° 0’ 24.737” S, 47° 22’ 52.941” W), PP3 (2° 59’ 56.264” S, 47° 22’ 17.735” W), PP4 (2° 58’ 56.895” S, 47° 21’ 37.938” W) and extending to the river outlet from the city of Paragominas at PP5 (2° 57’ 54.169” S, 47° 20’ 15.140” W). In the case of the Cereja River, samples were collected using a similar design as previously described, the pristine point was BP1 (1° 4’ 5.970” S, 46° 47’ 28.302” W), followed by BP2 (1° 3’ 23.711” S, 46° 46’ 36.339” W), BP3 (1° 3’ 13.535” S, 46° 46’ 17.706” W), BP4 (1° 3’ 3.126” S, 46° 45’ 54.696” W) and BP5 (1° 2’ 53.503” S, 46° 45’ 35.547” W).

### Genetic material and sequencing

In the laboratory, the membranes stored in the 50 mL Falcon tubes were thawed and washed directly inside their storage tubes using the same TSE buffer in which they had been preserved; washing was performed by repeatedly aspirating and dispensing the buffer over the membrane with a pipette to resuspend the retained particulate material. Genetic material was extracted using the Pro-DNeasy PowerSoil kit (Qiagen) according to the manufacturer’s instructions. The integrity of the obtained genetic material was assessed through horizontal electrophoresis. DNA concentration and purity were assessed using a NanoDrop spectrophotometer (Thermo Fisher Scientific), while DNA integrity was evaluated by agarose gel electrophoresis.

For the construction of DNA libraries, the samples were processed according to the manufacturer’s protocol for the Nextera XT DNA Library kit (Illumina). Sequencing was performed using the NextSeq 6000 System High-Output kit, generating paired-end reads of 150 bp, using the Illumina NovaSeq 6000 High-Output platform.

### Sequence analysis

The quality of the sequencing reads was initially analyzed using FASTQC software, version 0.11.9 [17]. Subsequently, the reads were trimmed and filtered using Fastp software [18] with a minimum Phred quality score of 20.

For bacterial diversity and composition analysis, the reads that passed the Quality control step were compared with the kraken2 software [19] using the AWS database index PlusPFP (Standard plus Refseq protozoa, fungi & plant) last updated in December of 2024. The obtained results were concatenated and converted, using Krakentools, to a format accepted by microeco [20], which had their composition analyzed by this package.

ARG genes were identified using the reads from each replicate using the CARD-RGI database [21]. The identified genes were filtered to retain those with at least 75% completeness. Subsequently, metadata corresponding to the “efflux pump” mechanism, “drug class,” and “gene family” were selected. Abundance values were normalized by generating a relative abundance calculated based on the aligned reads over the total reads present in each sample [22]. Genetic diversity values were also calculated using the Shannon diversity index with the PAST program.

Efflux pump repressor genes were selected from the CARD-RGI database and a literature review Gaetano et al. [7]. A list of genes involved in efflux pump gene repression was also extracted from NCBI databases (https://www.ncbi.nlm.nih.gov/) and amino acid sequences from the SWISS-MODEL Repository database [23].

### Alignment of repressor genes

The custom repressor protein database was used as an index for the Bowtie2 software [24]. Then, the reads were mapped against the database using three different strategies: (1) The reads from each replicate (30 samples) were mapped against the database; (2) The replicates from each point were concatenated, and the concatenated reads were mapped against the repressor protein database, generating the mapping result for each sample point (20 samples); (3) The reads from all samples were concatenated by city (5 samples) and mapped against the repressor protein database. In this way, different alignments were generated to analyze the sequence of the repressor proteins at progressively higher levels of completeness.

The data from each alignment were inserted into the SAMtools software [25] to calculate the number of aligned reads at each point with each of the repressor genes, and the gene coverage was calculated with the bedtools *genomecov* program present in the Bcftools package [26] and the alignments were visualized with Artemis [27].

### Modeling of the CbrB protein

For modeling the CbrB protein, the nucleotide sequence of the *cbrB* gene was translated into amino acids using a Python script (version 3.11.13) with the Biopython library. Undefined residues (’X’) in the protein sequence were replaced by alanine (’A’) to allow modeling. Homology modeling was performed using Swiss-Model software [28], using templates with an identity greater than 90% for high-coverage regions and at least 30% for low-coverage regions. RMSD calculation was performed using TMalign (v.20220412). To assess the presence of mutations in the models of each sample point, a Python (version 3.11.13) script with the BioPython and OS libraries was used.

### Statistical analysis

The data obtained from the different analyses were evaluated for normality using the Shapiro-Wilk test. Subsequently, they were analyzed using ANOVA or Kruskal-Wallis tests using the PAST 4.05 software [29]. The Orange Data Mining program [30], as well as the Seaborn and Vegan-Altair libraries of Python, were used to generate the graphs.

## Results

### Physicochemical parameters and sequencing overview

**Fig. 1:**
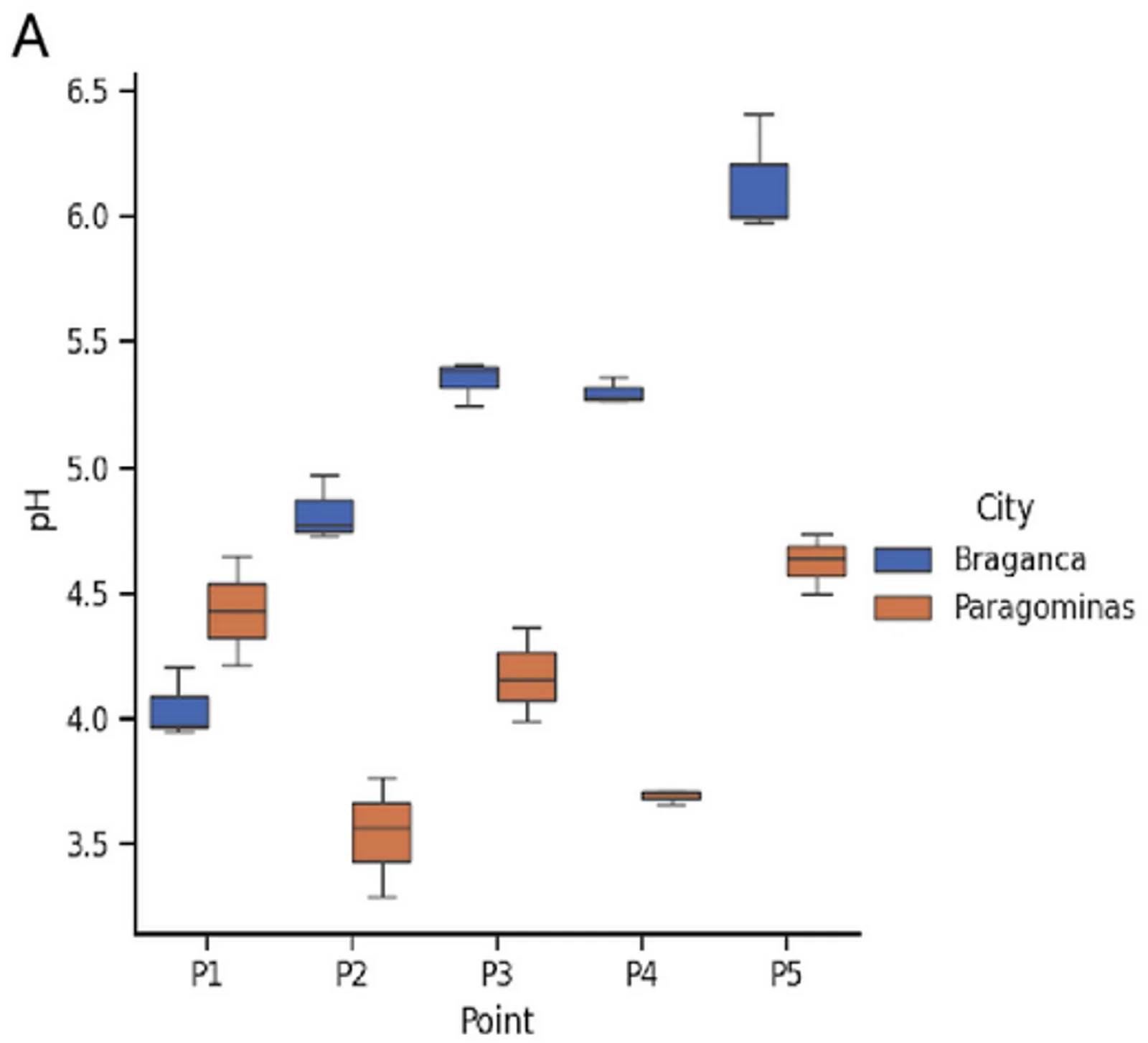
pH values in the municipalities of Bragança and Paragominas

The pH values obtained at the different points along the river’s courses showed acidic characteristics. In the Bragança area, values ranged between 3 and 6.4, with values of 4 at the pristine site, increasing up to point 5, and significant differences were observed between almost all points (ANOVA, F = 83.23; p-value = 1.23 × 10⁻⁷), with no significant difference between points 3 and 4 (Fig. 1). The municipality of Paragominas presented dispersed values among the points, with significant differences between them (ANOVA, F = 21.143; p-value = 7.2 × 10⁻⁵), particularly between P1–P2 (Tukey test; p-value = 0.001), P1–P4 (Tukey test; p-value = 0.003), P2–P3 (Tukey test; p-value = 0.009), P2–P5 (Tukey test; p-value = 0.000), P3–P4 (Tukey test; p-value = 0.045), and P4–P5 (Tukey test; p-value = 0.000) (Fig. 1).

The yield of the reads obtained from the sequencing of the different sampling points was, on average, 31,291,237 reads for the city of Bragança and 70,185,881 reads for Paragominas. These data allowed the identification of antimicrobial resistance genes.

### Bacterial composition and diversity

Alpha diversity analysis of the different points of the analyzed rivers showed high values of the Shannon diversity index, with values between 7.184 and 7.488 for the Uraim River in Paragominas [31]. For the Cereja River in the city of Bragança, values ranged between 6.728 and 8.409. No significant differences were found between the points of each city.

**Fig. 2:**
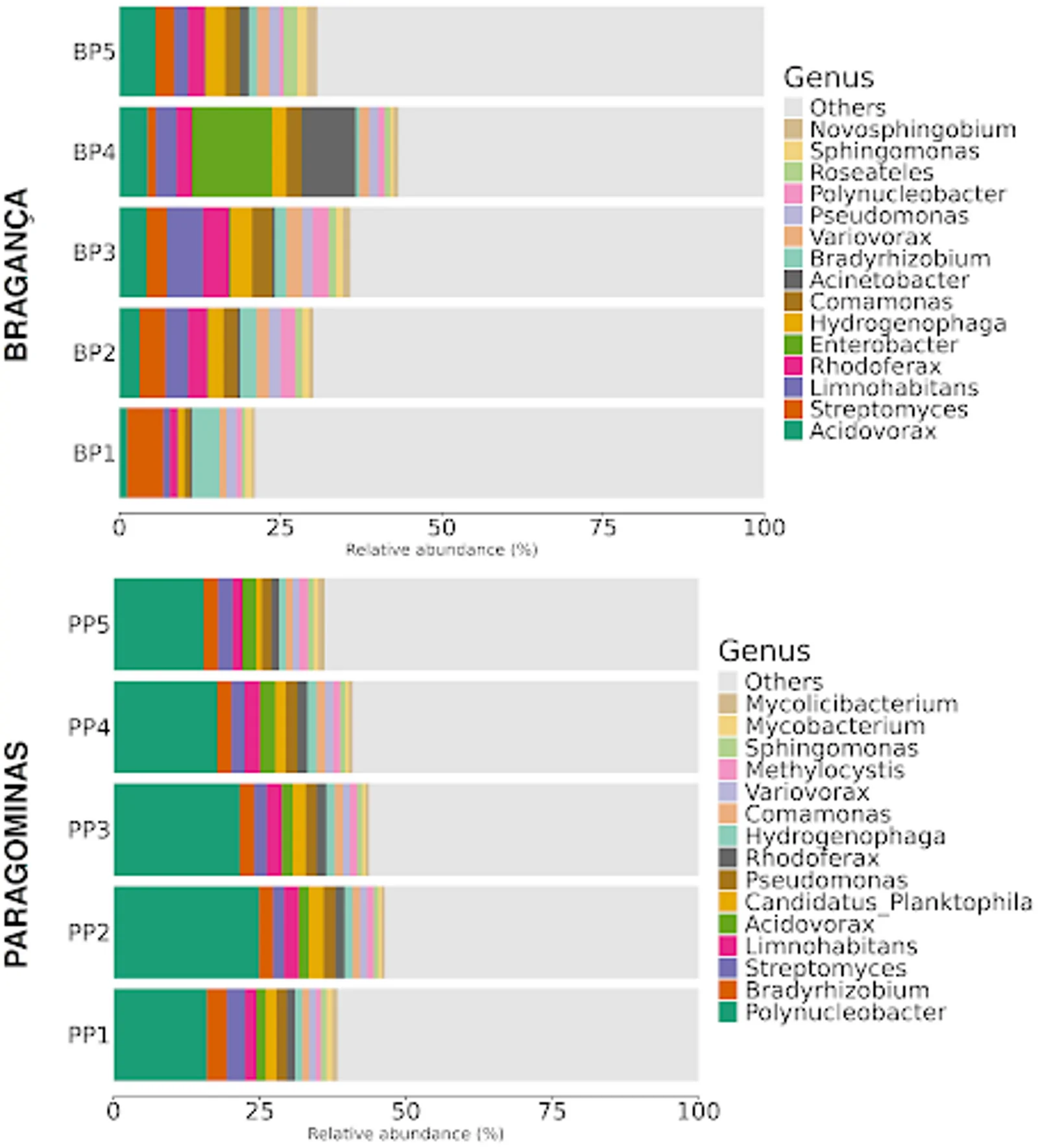
Relative abundance of the 15 most abundant genera identified in each city from the metagenomic data

Regarding the abundance composition at the genus level for the cities of Bragança and Paragominas, the 15 most abundant genera from each city were selected (Fig. 2). In the city of Bragança, the most prevalent genus was *Acidovorax*, which increased from the pristine point to the most polluted one; conversely, the presence of *Streptomyces* decreased. Likewise, *Enterobacter* stood out in BP4, and *Acinetobacter* showed a higher relative abundance (Fig. 2). In the Paragominas river, the most prevalent genera were *Polynucleobacter*, followed by *Bradyrhizobium*,* Streptomyces*, and *Limnohabitans* (Fig. 2).

**Fig. 3:**
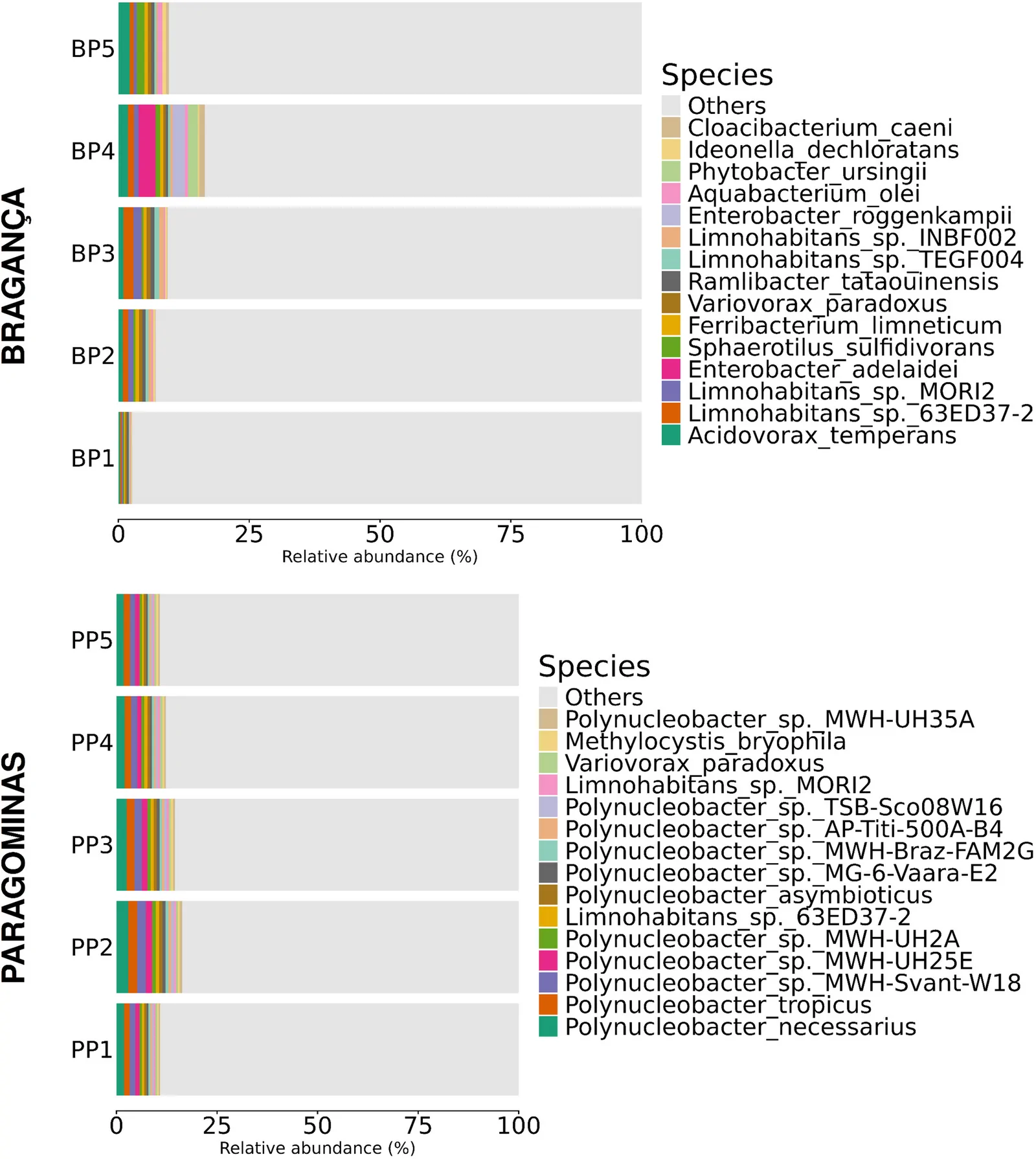
Relative abundance of the 15 most abundant species identified in each city from the metagenomic data

At the species level (Fig. 3), the distribution was relatively balanced, with no single species showing a marked predominance. In Bragança, the most notable result was observed at point BP4, where Enterobacter adelaidei showed greater relative abundance, followed by *Enterobacter roggenkampii*. In Paragominas, this pattern was observed more generally across the sampling points, with no individual point showing a marked predominance. Instead, greater representation was observed for a group of species within the genus *Polynucleobacter*, including *P. necessarius*,* P. tropicus*,* P. sp. MWH Svant W18*,* P. sp. MWH UH25E*, and *P. sp. MWH UH2A.*

### Antimicrobial resistance genes (ARGs)

#### Diversity and composition

Regarding the diversity of antimicrobial resistance genes in the municipalities, the Shannon genetic diversity values of the ARGs from the pristine and the more polluted points in Bragança were similar, except at point 4, which showed the highest diversity and presented significant differences (Kruskal-Wallis; *p-value* = 0.038) between points P1 and P4 (Dunn; *p-value* = 0.026). In the municipality of Paragominas, an increase in diversity values was observed from the pristine point up to the highest value at point 5, with no significant differences between the points. Comparisons between the two municipalities revealed differences at P2 (t-test; p-value = 0.021), P3 (t-test; p-value = 0.015), and P4 (t-test; *p-value* = 0.001) (Supplementary Material, SFigure 1).

**Fig. 4:**
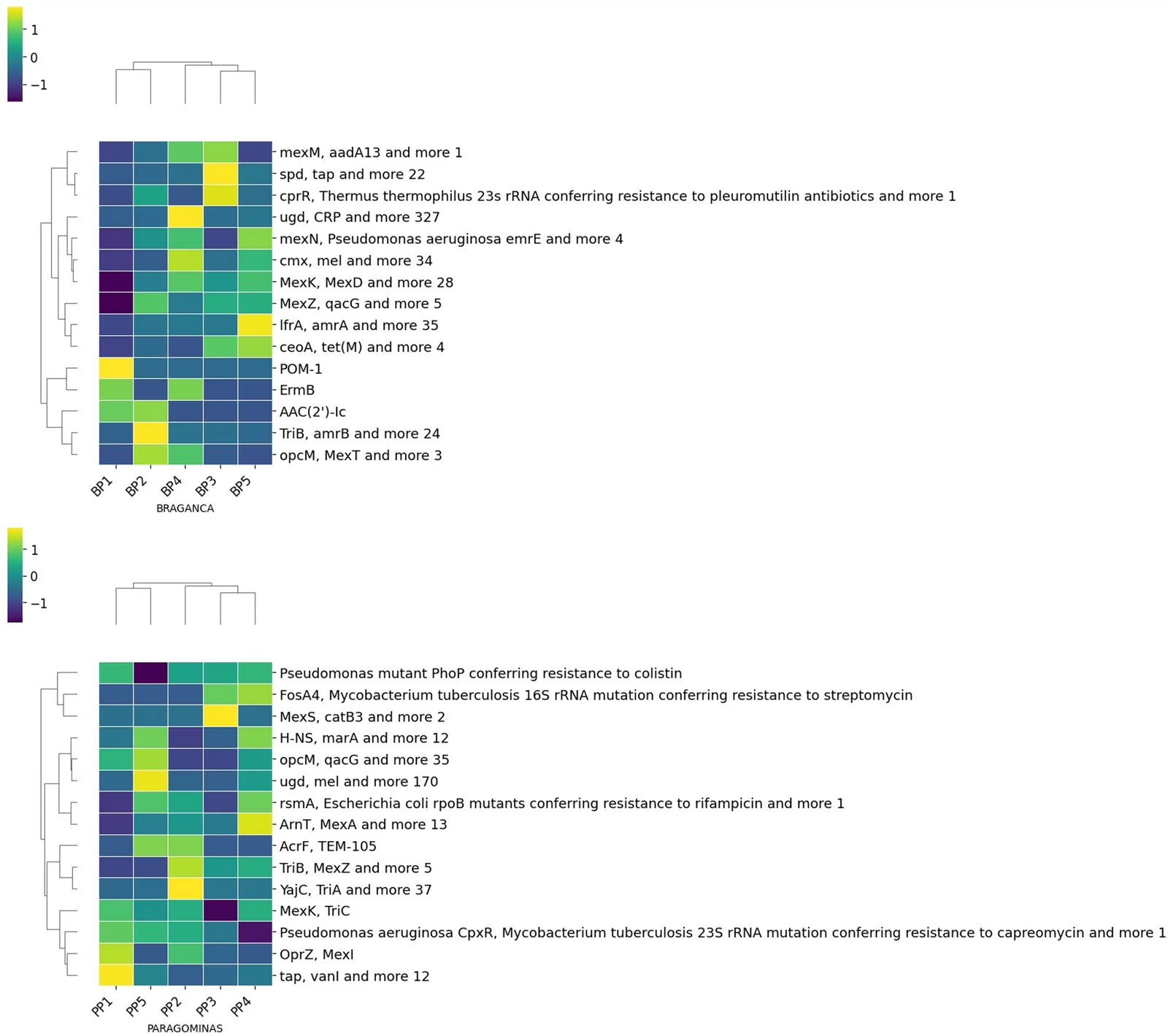
Heatmap showing the distribution of the most abundant antimicrobial resistance genes (ARGs) identified in the CARD-RGI analysis in the cities of Paragominas and Bragança. Gene abundances were normalized using Z-score transformation

Regarding the ARGs identified in the CARD-RGI analysis (Fig. 4), Bragança showed higher abundances of genes such as *spd*,* cprR*,* ugd*,* POM-1*,* lfrA*,* TriB*, and *amrAB*, among others. In Paragominas, the most prominent genes included *MexS*,* catB3*,* ugd*,* mel*,* arnT*,* MexA*,* yajC*,* TriA*,* tap*, and *vanI*, among others. Most of the identified genes were associated with resistance mechanisms involving efflux pumps, although other mechanisms of antimicrobial resistance were also represented.

#### Mechanism of resistance

**Fig. 5:**
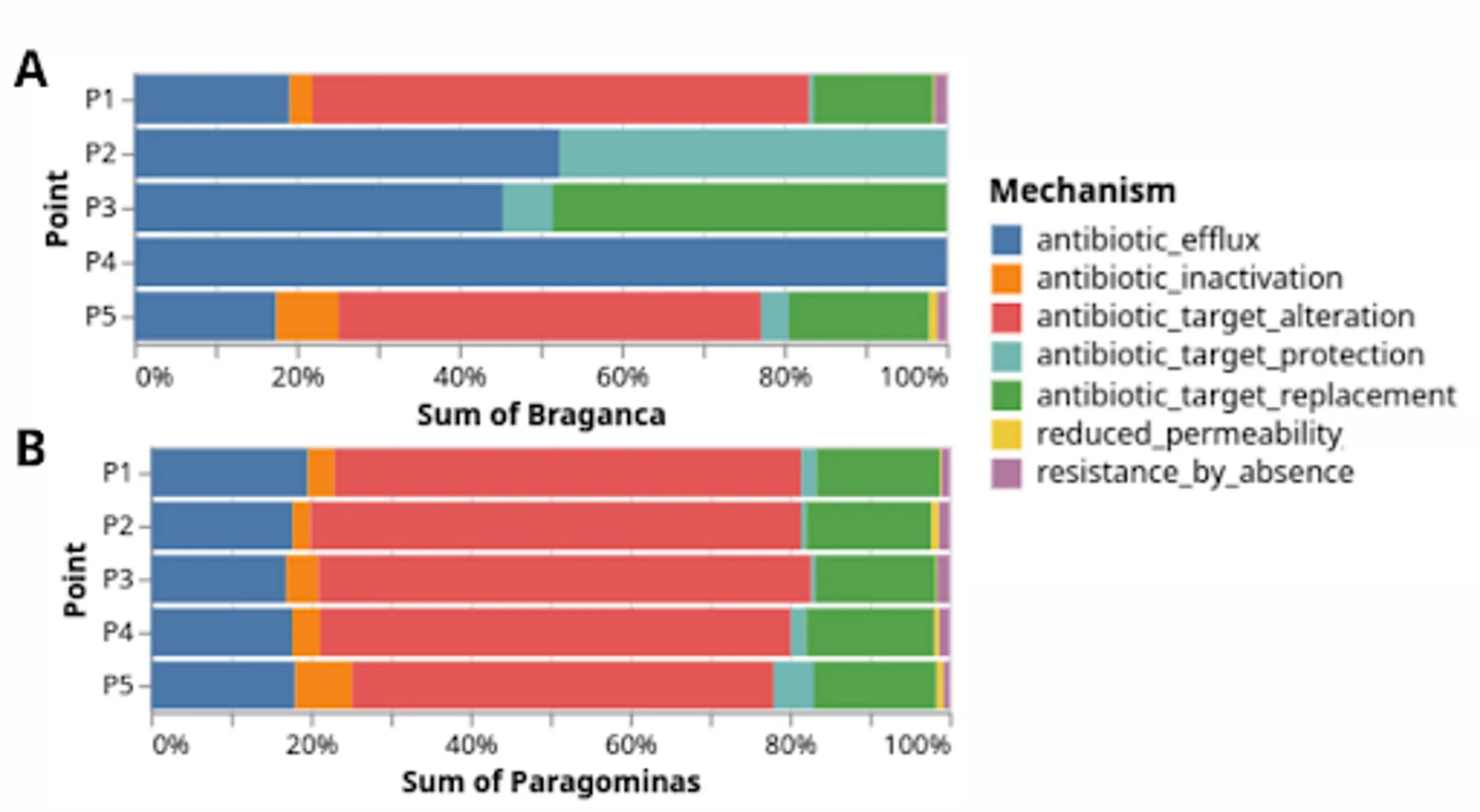
Distribution of resistance mechanisms that correspond to the different ARGs of the cities (A) Bragança and (B) Paragominas

The identified ARGs exhibited distinct distribution profiles of resistance mechanisms between the cities. In Bragança, point 4 showed the highest abundance of efflux pump mechanisms, whereas antibiotic target-shift mechanisms were found at the pristine points, with the lowest abundance (Fig. 5A). In Paragominas, the different points were similar to one another, with a predominance of antibiotic target-shift mechanisms and a smaller proportion of efflux pumps (Fig. 5B).

### Efflux pumps (EP)

The identified efflux pump genes exhibited low genetic diversity values at the pristine sites, with an increase in diversity along the rivers’ course through the cities. Point 4 in Bragança was particularly noteworthy, showing significant differences (ANOVA; *p-value* = 5.045 × 10⁻⁹) between points P1 and P4, and between P4 and all the other points (Tukey test; *p-value* = 0.000), as well as between points P2 and P5 (Tukey test; *p-value* = 0.042). In Paragominas, no significant differences could be identified between the points. These EP genes have the potential to confer resistance to up to 15 different drug classes, with relevance at various points for resistance against one to three drug classes in the different municipalities, particularly in the city of Paragominas. In the municipality of Bragança, the EPs with the greatest resistance capacity covered between 10 and 15 different classes of drugs, at points 4 and 5 (*Supplementary Material*, SFigure 2AB).

**Fig. 6:**
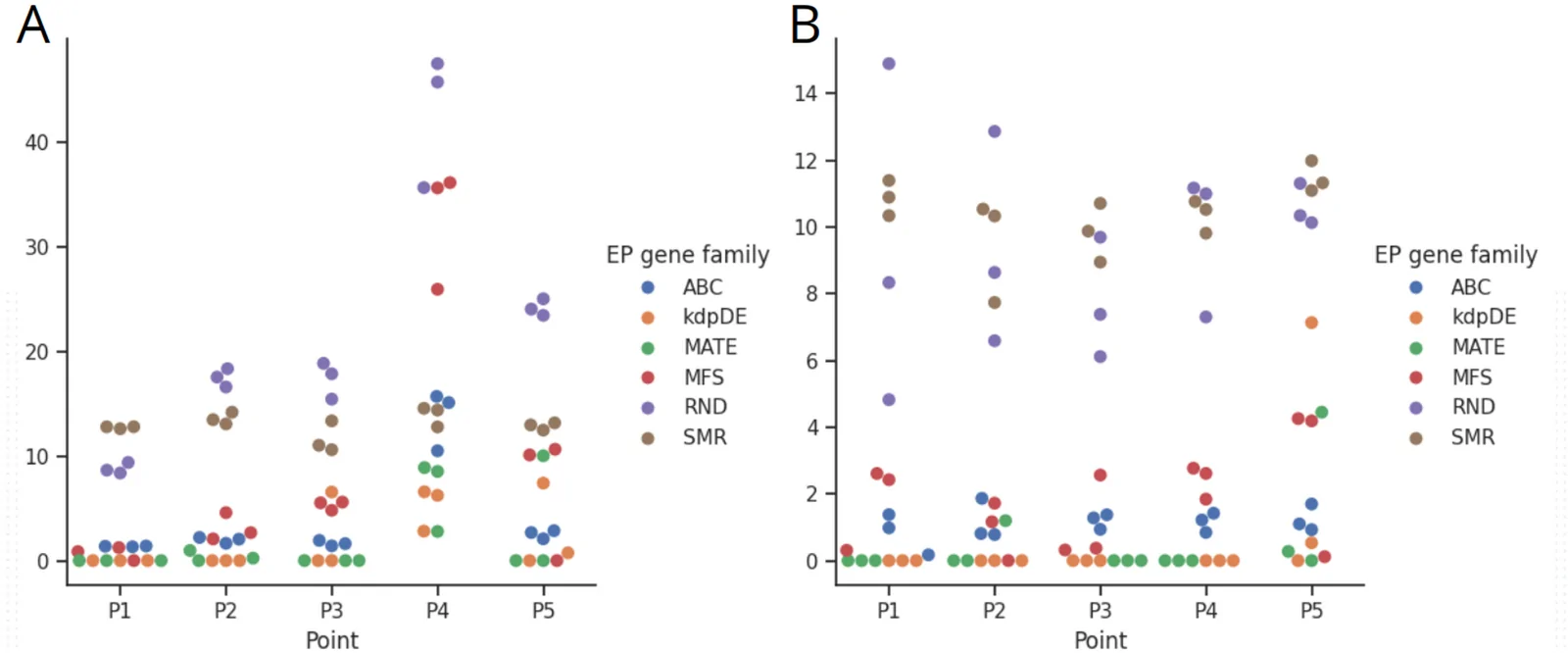
Composition of the different families that make up the flow pumps in the different cities, (A) Bragança and (B) Paragominas. ATP-Binding Cassette (ABC) superfamily, Multidrug and Toxic Compound Extrusion (MATE) superfamily, Major Facilitator Superfamily (MFS), Resistance Nodulation and Cell Division (RND) superfamily, and Small Multidrug Resistance (SMR) superfamily

The types of gene families identified (Fig. 6) with relevant abundance were the *RND* and *SMR* families in both municipalities, with particular relevance at point 4 of Bragança (ANOVA; *p-value* = 9.64 × 10⁻⁷), as well as the *MFS* (ANOVA; *p-value* = 6.6 × 10⁻⁶) and *ABC* (ANOVA; *p-value* = 1.3 × 10⁻⁶) families, with significant differences between point P4 and the others (Tukey test; *p-value* = 0.000) for the families indicated above (Fig. 6A). In Paragominas, however, the distribution of the RND and SMR families was similar (Fig. 6B).

In the distribution of genes by abundance in each municipality, the most abundant was *qacG*, which corresponds to the SMR family and is involved in resistance to disinfectant and antiseptic compounds. It was followed by the *adeF* and *MuxC* genes; the *mdtB* and *mdtC* complex, which is involved in resistance to the aminocoumarin, tetracycline, fluoroquinolone, monobactam, and macrolide drug classes; and the *OprM* gene, which is related to resistance to 15 different drug classes and corresponds to the RND family.

**Fig. 7:**
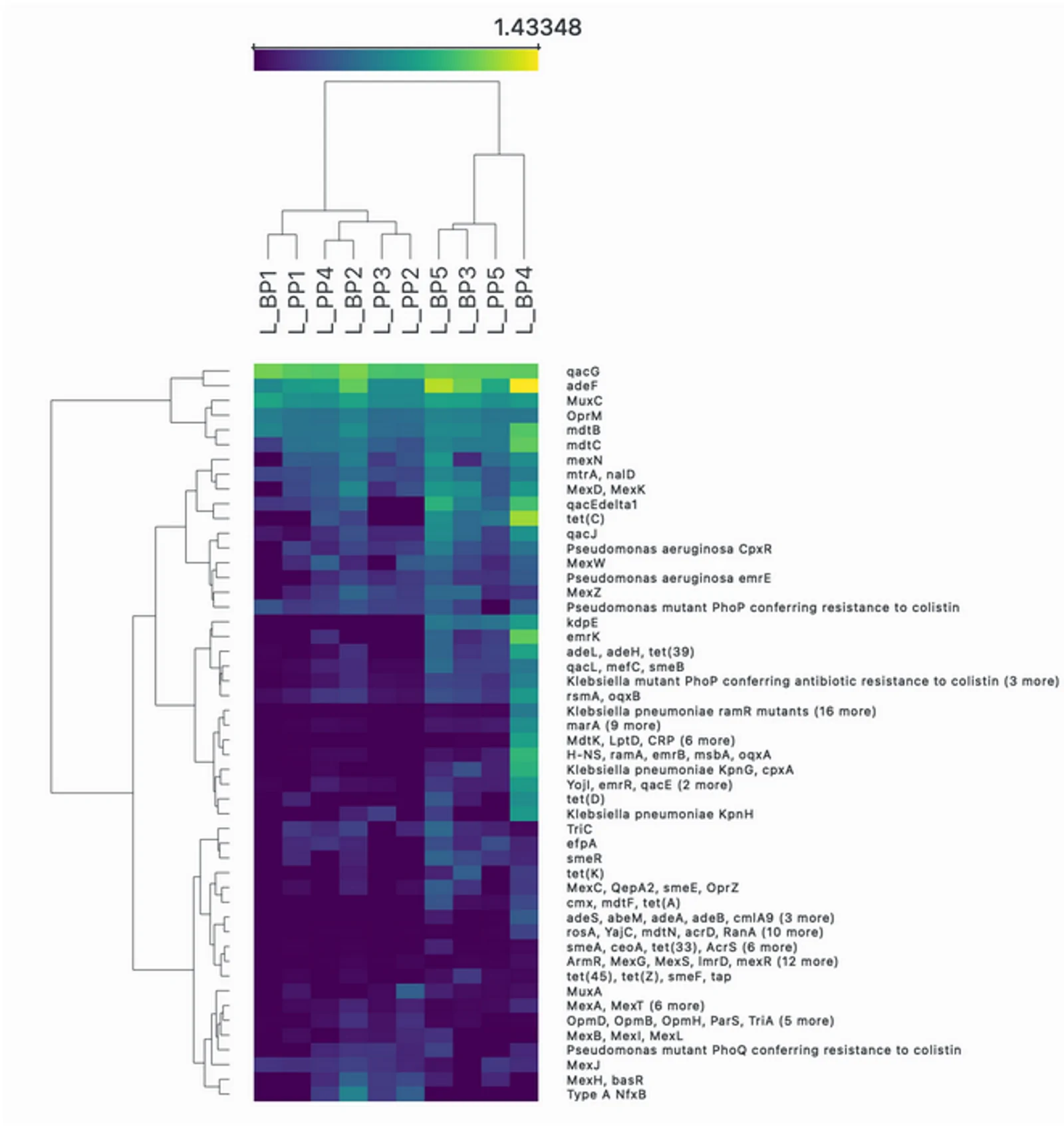
Heatmap arrangement of the different EP genes identified with the CARD RGI database of the cities under study. The abundance values were transformed with logarithms (log (*n* + 1)), and the points were presented as BP# corresponding to the city of Bragança and PP# to the city of Paragominas

The grouping of the different collection points from the cities revealed two clusters: in the first, the pristine sites (BP1 and PP1) stood out, together with most of the sites from the city of Paragominas; in the other cluster, the presence of the sites BP3, BP4, and BP5 in the city of Bragança and PP5 in the city of Paragominas was significant (Fig. 7). Point 5 in Bragança showed a decrease in the abundance and diversity of these genes.

### Repressors identified

**Fig. 8:**
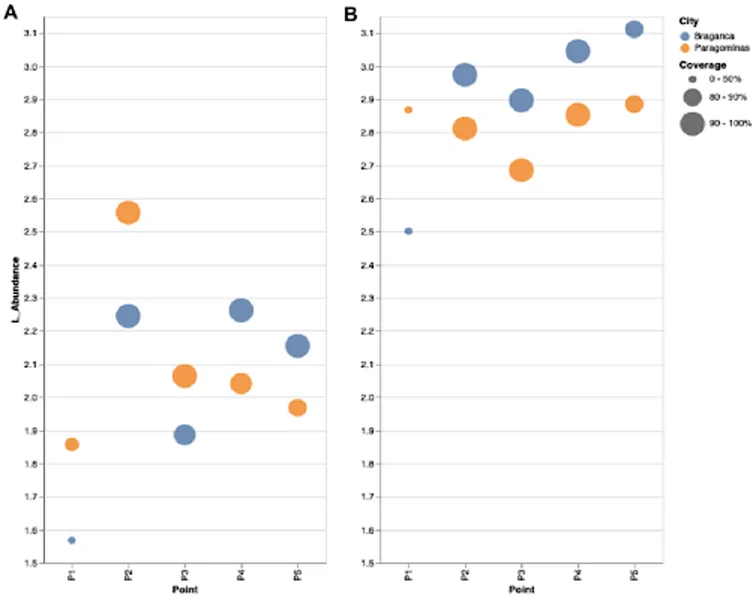
Distribution of alignments of reads of the repressor genes (A) *cbrA* and (B) *cbrB* identified at the different collection points in the two municipalities

Regarding the genes involved in EP repression, the most abundant in both municipalities were the cbrB and gacS genes, which comprise the TCS family. When the abundances of the different genes at the different collection points were clustered in the heatmap, points 5 and 1 — corresponding to the most polluted and the pristine sites, respectively — were distinguished. A higher abundance of genes was also observed at point 4 in the municipality of Bragança, distinguishing its clustering from that of the other points (*Supplementary Material*, SFigure 3). The alignment of these genes highlighted that the genes with the highest coverage at almost all points correspond to the TCS group, such as the *cbrAB* genes, and their abundance was lower, with cbrA being less abundant than the *cbrB* gene (Fig. 8).

### *cbrB* repressor gene and Modeling protein

The alignment of the reads from the different points in the cities with the Pseudomonas aeruginosa genome sequence of the *cbrB* gene (AB045630.1:4373–5809) revealed the presence of this gene at all sampling points, with different levels of coverage (region of the gene that had at least two aligned reads) and sizes between 883 and 1438 nt. At the remaining points, this gene was identified with better coverage, especially at point 2, with 1437 nt, and with contents of “N” nucleotides of 0.7 and 0.84%.

The multiple alignment of the recovered sequences of this gene made it possible to identify two groups: one with low coverage, corresponding to points 1 and 5, and another with better performance, grouping points 2, 3, and 4 (*Supplementary Material*, SFigure 4 A). The multiple sequence alignment of the *cbrA* gene showed groupings similar to the previous one (*Supplementary Material*, SFigure 4B); nevertheless, this gene did not present low coverage values at the different points.

**Fig. 9:**
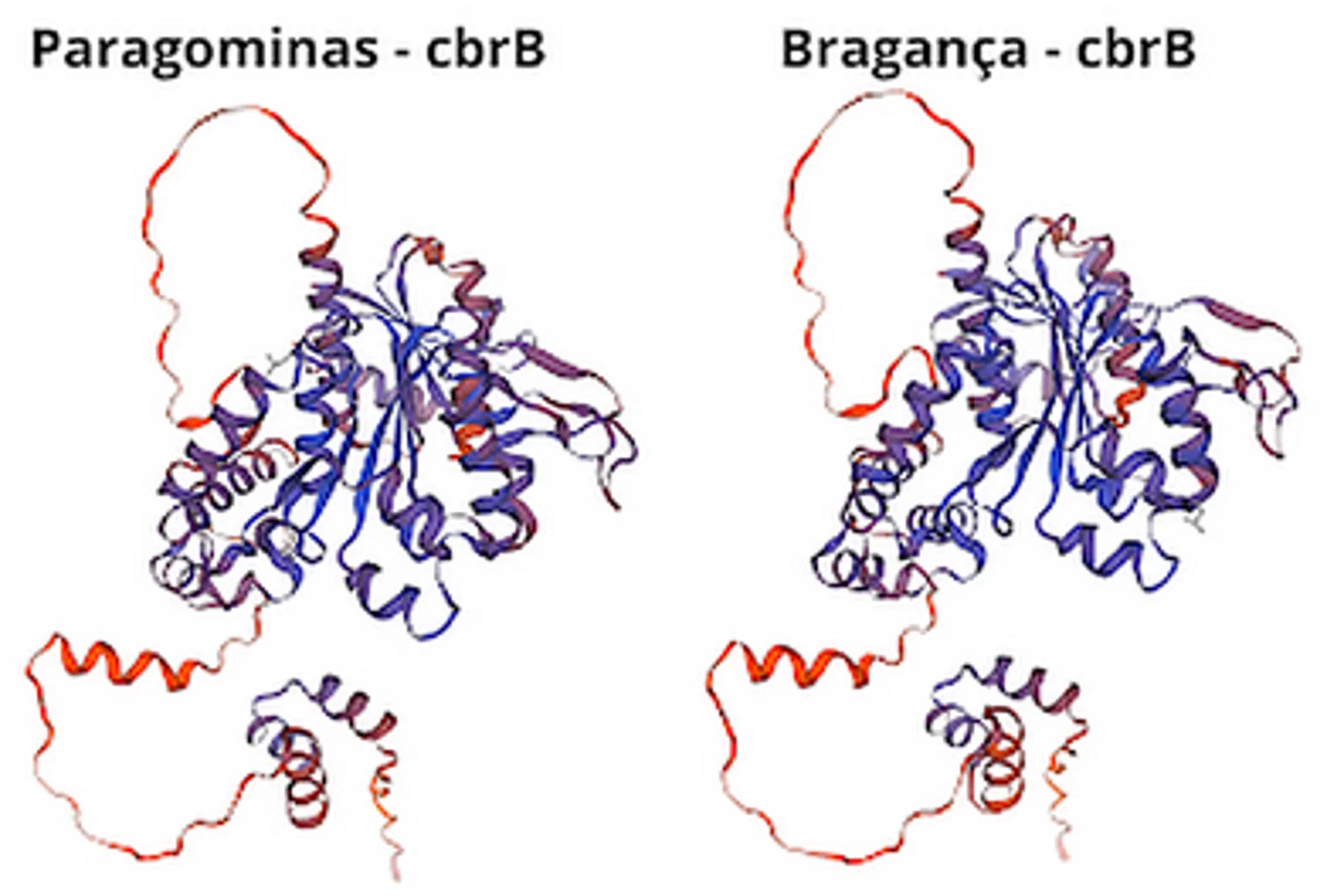
CbrB protein modeled from the consensus nucleotide sequence from the cities of Bragança and Paragominas, using the structure of the AlgB protein (ID Q9HV73.1.A) as a template. For the Paragominas cbrB, the indices were GMQE = 0.81, Seq. ID = 97.28%, and Coverage = Complete. For the Bragança cbrB, the indices were GMQE = 0.81, Seq. ID = 96.65%, and Coverage = Complete

To perform the modeling, the nucleotide sequence of the *cbrB* gene (NCBI ID: BAB20867.1) was used, which showed 100% identity with the Alginate biosynthesis transcriptional regulatory (AlgB) protein, ID Q9HV73.1.A. With the consensus sequences obtained from the cities of Bragança and Paragominas, protein modeling was performed (Fig. 9). These sequences present 96.65% and 97.28% identity, respectively, with the AlgB protein; therefore, they were considered as templates for protein modeling in both cases.

The sequence characteristics obtained from the cities presented a GMQE index identical to that of the model sequence, with a value greater than 0.8 for both cities, indicating a good quality of the obtained models. Stereochemical analysis using the Ramachandran plot revealed that over 90% of the residues are located in energetically favored regions, reinforcing the coherence of the structure (*Supplementary Material*, SFigure 5).

Comparison of the consensus sequence of the cities with the NCBI *cbrB* sequence using the RMSD index confirms high structural similarity (RMSD = 0.01) to the template. Furthermore, the QMeanDiscoGlobal index of Bragança and Paragominas (0.67 ± 0.05 and 0.66 ± 0.05, respectively) is comparable to that of the NCBI model (0.65 ± 0.05).

Sequences were constructed for each of the sites, indicating greater conservation of the protein structure in PP2 and PP3 from Paragominas and in BP2 from Bragança, with greater than 90% identity to the AlgB protein (Supplementary STable 1).

The analysis of mutations in known regions of the AlgB protein, carried out to determine the stability of this sequence, indicates the regulatory domains of the CbrB protein from the different points of the cities under study, which present two domains that perform regulatory functions. Of these, the regulatory response domain (3-113) was considerably conserved for the city of Bragança, with some substitution and deletion mutations. In Paragominas, sequence variations occurred at all sites, particularly sites 3 and 4 (*Supplementary Material*, SFigure 6 A). The Sigma 54 (149–378) domain presents a situation similar to that previously described, with point mutations at site 5 in the Bragança River and in Paragominas; however, the sites containing the highest number of mutations were sites 3 and 4 (Supplementary Material, SFigure 6B).

## Discussion

### pH and water quality

The municipalities present different economic activities, which may be reflected in the physical and chemical characteristics of the water bodies. The pH values differ between the two cities, as these water quality data suggest that water acidity is linked to soil characteristics [32]. In the case of the Cereja River, the results reveal that human activity has significantly influenced water quality, as indicated by the increase in values compared to those found at the pristine site.

The acidic water characteristic recorded at the headwaters of the analyzed rivers is typical of Amazonian rivers and streams that drain flooded forest areas, which present high concentrations of humic and fulvic acids [33]. The loss of riverside vegetation cover is associated with increased temperatures, reduced organic matter, decreased oxygen levels, and consequent changes in biodiversity [34].

### Efflux pumps as resistance and adaptation mechanisms

Efflux pumps are protection mechanisms against different compounds, allowing bacteria to adapt to the different environments that can be colonized by them [35]. In addition to acting as antibiotic resistance mechanisms in the environment, they expel toxic substances such as heavy metals, biocides and quorum sensing molecules (autoinducers), biofilm-forming molecules, and bacterial virulence factors [5]. These mechanisms are frequent in Gram-negative bacteria, as well as in *Escherichia coli* [36], which increases the chance of co-selection of multidrug-resistant bacteria [37].

In the Uraim River, the noticeable influence of anthropogenic actions, such as deforestation and population increase, has been reported on both the diversity and the prevalence of pathogenic bacterial strains, with emphasis on *Enterobacteriaceae* and members of the ESKAPE group [33], which may carry these resistance mechanisms in their genomes.

### Diversity of efflux pumps and horizontal gene transfer

The genetic diversity of efflux pump molecules can be acquired through horizontal gene transfer (HGT), influencing the presence of different types of molecules in these mechanisms, which constitute an adaptation mechanism for bacteria [38]. This could explain the increased diversity values of the most polluted sites compared to the pristine site, since this gene transfer is more active in contaminated water bodies, ensuring the transfer of bacterial resistance [39]. Likewise, the expression of EP genes can also be altered by mutations that can lead to the overexpression of efflux pumps [40].

The increase in various anthropogenic pollutants has led to the emergence of different molecules that diversify bacterial adaptation mechanisms. EPs can confer resistance to other compounds, such as heavy metals and organic pollutants, and may have relevant functions for bacterial behavior in natural environments, thus being significant for bacterial physiology and its evolution in natural ecosystems [41]. Therefore, efflux pumps not only contribute to multidrug resistance but also to biofilm formation, virulence, and pathogenesis [42].

### Gene families

The RND family is frequently present in Gram-negative bacteria [1]. This type of family demonstrates broad substrate specificity for structurally diverse molecules, such as antibiotics, anticancer agents, dyes, bile salts, detergents, and solvents [7], and is also involved in the dissemination of determinants in pathogenic processes in *E. coli* and *P. aeruginosa* [43, 35], and especially in the adaptation to changes in physicochemical characteristics [36].

The greater abundance of EPs identified in these municipalities, where there is greater anthropogenic activity, may suggest an increase in the copies of these genes, allowing the adaptation of bacteria to these ecological niches as a response to stressful components of the environment [44, 45]. It should also be considered that the presence of these molecules ranges from 6 to 18% of the proteins in the extracellular membrane of bacteria [2].

In this sense, point 4 in Bragança stands out for its greater abundance and diversity of EP genes, which may indicate a greater load of pollutants from different sources in this segment of the river, affecting the ecological niche. The decrease at point 5 in the same city may be influenced by the Caeté River encroaching on the Cereja River, diluting pollutants and microorganisms.

### Repressors and the TCS system

The TCS system comprises at least two essential proteins: a membrane-bound dimeric histidine kinase subunit, a transmembrane domain (TMD), one or more signal transduction units, and a response regulator [46]. This is one of the main signal transduction mechanisms for responding to environmental cues [47]. The presence and response of these molecules to environmental variations could be considered not only protective against MDR bacteria, but also indicators of anthropogenic impact.

The different families of EPs identified are involved in the dissemination of MDR bacteria. However, the presence of inhibitory or repressive molecules that regulate the expression of these bacterial components can, through different inhibition mechanisms, interfere with the efficiency of EPs [7, 48], reducing minimum inhibitory concentration (MIC) values and antibiotic resistance [7, 35]. These mechanisms that affect EPs are interesting targets for study to combat antibiotic resistance [44]. Furthermore, it was found that inhibition of EP function affected some virulence factors related to QS (*quorum sensing)*, as well as cell motility [48, 20].

The *cbrAB* genes were identified as repressors activated by various environmental signals, such as host receptors, antimicrobials, divalent ions, organic solvents, pH, and oxidative stress [7, 35]. This could explain the increased abundance of these genes at sites with greater anthropogenic pollution in both municipalities. Nevertheless, the increased abundance of these types of genes is a response to the stress suffered by microorganisms in the ecosystem [45]. Due to the rate of duplication and genomic amplifications present in each bacterium (approximately 10%) as mechanisms of bacterial adaptation [45], this would justify the greater abundance, especially of the *cbrB* gene, which may play an important role in the regulation of different genetic targets, in addition to the response of resistance to antimicrobials, as was observed in the regulation of efflux pumps in the pathogen *Acinetobacter baumannii* [49].

### cbrAB, protein modeling, and mutations

The group of cbrAB repressor genes of the TCS family, involved in nutritional adaptation processes, was initially described in *Pseudomonas aeruginosa* as a regulatory system involved in the hierarchical utilization of various carbon sources. In addition to its relevant role in catabolic repression, it also modulates processes such as biofilm formation, production of pyocyanin and rhamnolipids, *quorum sensing* (QS), secretion of protein virulence factors, and antibiotic resistance [47].

This system comprises the CbrA protein, a kinase sensor [47], and the CbrB protein, a transcriptional activator of RNA polymerase [50], which has different targets for protein expression, including the RND family efflux pumps [51].

The relevance of this gene would justify its better representation at the different points, making it possible to recover the partial sequence of the *cbrA* gene and other repressor genes, which could indicate the *cbrB* gene as an alternative marker to evaluate, as it may be involved in different cellular processes of the bacteria to adapt to the increase in pollutants in the environment.

Although there are variations between the sequences, the structural quality of the model remained conserved, suggesting that the protein modeled using metagenomic sequence data retrieved from this type of regulatory protein — although the CbrB protein is not included — requires further analysis to assess the conservation of the protein’s functional sites.

Considering the known roles of the *cbrAB* two-component system in *Pseudomonas aeruginosa* metabolism, its ability to regulate the expression of some AlgB protein synthesis genes was identified in *Azotobacter vinelandii* [52]. This corroborates the identity obtained between the recovered gene sequence and the Q9HV73.1 template, suggesting that this system may be related to multiple stress resistance mechanisms. Furthermore, the greater coverage of *cbrB* observed at some sites may reflect the greater need for gene duplication as an adaptive measure for bacteria to cope with environmental stress [42], thus reinforcing the potential application of *cbrB* as a marker of environmental pollution.

Meanwhile, the models developed for the point sequences of each city differ from the model used as a template, even considering the biases of the sequencing methodology [53]. In addition, there were different sequencing yields for genes with divergent gene coverage, similar to point 1.

The regulatory response domain (3–113) was considerably conserved in Bragança, although some substitution and deletion mutations were observed, which may be influenced by sequencing artifacts. This may reflect the presence of distinct sequences, either due to the contribution of different bacterial communities, a high mutation rate influenced by hospital waste (especially at site 3), or the influence of sequencing. Sigma 54 is associated with the transcriptional regulation of genes such as virulence genes in pathogenic species [54].

### Study limitations

Some limitations of this study should be considered when interpreting the results. First, efflux pump repressor genes were identified through homology-based database curation (CARD-RGI, NCBI) and read mapping, rather than through direct functional or experimental validation of repressor activity; therefore, the regulatory role inferred for genes such as *cbrB* reflects sequence-based evidence rather than confirmed gene expression or repression capacity. Second, sampling was carried out at a single time point in each river, which precludes an assessment of seasonal or hydrological variation (e.g., dry versus rainy season, discharge and dilution effects) on the abundance and diversity of efflux pump and repressor genes. Third, the recovery of the *cbrA* and *cbrB* genes showed lower coverage at points 1 and 5 in both rivers, which limited the resolution of the mutation analysis at these sites, particularly within the Sigma 54 domain. Future studies incorporating multiple sampling campaigns, targeted deep sequencing of low-coverage regions, and functional assays (e.g., gene expression or knockout analyses) would help validate and extend the findings reported here.

## Conclusion

Different profiles of antimicrobial resistance mechanisms were identified in the Cereja and Uraim rivers, highlighting an increase in the diversity of genetic components related to efflux pumps in areas where pollutants are more abundant in the rivers. The most abundant gene families were RND and SMR, with resistance to up to fifteen different antibiotics.

Furthermore, the presence of repressor genes involved in regulating efflux pump (EP) genes was characterized, with a greater prevalence of genes corresponding to the TCS family being identified, particularly the *cbrAB* genes, which are involved in regulating EPs and aiding bacterial adaptation. The sequence of the *cbrB* gene was recovered, enabling the modeling of the protein in the two cities analyzed, especially in places with greater pollution.

Characterizing EP and repressor genes in ecosystems allows us to visualize the bacterial adaptation response to anthropogenic impact. In this work, we successfully identified multidrug resistance genes for antibiotics and biocides in bacterial communities, as well as the presence of repressor genes that may be useful for controlling multidrug-resistant strains and may serve as an alternative biomarker of environmental stress.

Despite limitations related to functional validation, single-time-point sampling, and the coverage of some regions of the cbrA/cbrB genes, this study represents an initial approach to expanding our understanding of the dynamics of resistance and regulatory genes in Amazonian aquatic environments through metagenomic data. These findings may provide a basis for future investigations, particularly those focused on functional validation and monitoring of these genes under different environmental conditions.

## Supplementary Information

Below is the link to the electronic supplementary material.

Supplementary Material 1

## Data Availability

The data presented in the study was deposited in the National Center for Biotechnology Information NCBI database (https://www.ncbi.nlm.nih.gov/), BioProject ID PR JNA1122411.
