# Supplementary material for: Prospecting for efflux pump and repressor genes in rivers of the Amazon region of Brazil
Source: Mol Biol Rep. 2026 Oct 23;53(1):1800. doi: 10.1007/s11033-026-12979-1 (PMC13694302; doi:10.1007/s11033-026-12979-1)
Supplement: Supplementary file 1 [file 11033_2026_12979_MOESM1_ESM.docx]

**MATERIAL SUPPLEMENTARY**

**Supplementary material Sfigure 1:** Box plot of Shannon genetic diversity values of antibiotic resistance genes in the cities of Bragança and Paragominas

Figura 2.

**Supplementary material Sfigure 2:** Distribution of the number of different drug classes of the flux pump genes identified in the city A. Bragança and B. Paragominas.

A

B

**Supplementary material Sfigure 3:** Heatmap arrangement of the different EP repressor genes identified in the cities under study. Abundance values were logarithmically transformed [log(n+1)], and the points were presented as: BP# corresponds to the city of Bragança and PP# to the city of Paragominas.

**Supplementary material Sfigure 4:** Phylogenetic tree with the UPGMA method of multiple alignment of *CbrB* gene sequences recovered at different points in the municipalities of Bragança (*Cbr*-BPB#) and Paragominas (*Cbr*-PP#).

A

B

**Supplementary Material Figure S5**: Ramachandran plot of proteins modeled from the Paragominas and Bragança sequences, indicating the distribution of residues according to their energetic favorability.

**Supplementary Material STable** 1: Metrics for the structural quality of the cbrB protein modeled from reference sequences and recovered from metagenomic data.

| **Sample** | **GMQE** | **Seq. ID** | **Coverage** | **Ramachandran Favoured** | **QMeanDisco Global** | **RMSD CbrB** | **RMSD City** | **Template** |
| --- | --- | --- | --- | --- | --- | --- | --- | --- |
| NCBI seq | 0.81 | 100% | Complete | 93.07% | 0.65±0.05 | - | - | Q9HV73.1.A |
| Bragança* | 0.81 | 96.65% | Complete | 93.07% | 0.67±0.05 | 0.01 | - | Q9HV73.1.A |
| Paragominas* | 0.81 | 97.28% | Complete | 93.07% | 0.66±0.05 | 0.01 | - | Q9HV73.1.A |
| PP1 | 0.25 | 79.41% | Incomplete | 96.88% | 0.67±0.12 | 0.13 | 0.13 | A0A2D8ABY5.1 |
| PP2 | 0.81 | 98.33% | Complete | 93.07% | 0.67±0.05 | 0.01 | 0.00 | Q9HV73.1.A |
| PP3 | 0.81 | 93.93% | Complete | 93.49% | 0.64±0.05 | 0.05 | 0.05 | Q9HV73.1.A |
| PP4 | 0.53 | 82.99% | Incomplete | 96.61% | 0.64±0.05 | 3.88 | 3.88 | A0A1E4WJ64.1.A |
| PP5 | 0.40 | 100% | Incomplete | 91.38% | 0.40±0.11 | 04.06 | 4.06 | 7am2.70.A U4 |
| BP1 | 0.19 | 100% | Incomplete | 95.06% | 0.29±0.09 | 4.78 | 4.78 | 6t9i.1.L |
| BP2 | 0.81 | 97.70% | Complete | 93.07% | 0.67±0.05 | 0.01 | 0.01 | Q9HV73.1.A |
| BP3 | 0.65 | 74.51% | Incomplete | 95.41% | 0.58±0.05 | 3.74 | 3.74 | A0A5J6R7V6.1.A |
| BP4 | 0.80 | 81.68% | Incomplete | 93.87% | 0.60±0.05 | 0.02 | 0.02 | Q9HV73.1.A |
| BP5 | 0.48 | 33.88% | Incomplete | 94.85% | 0.56±0.05 | 3.65 | 3.65 | 5m7n.1.A |

*Consensus sequence of city points.

**Supplementary Material SFigure 6:** Number of real mutations, not related to the substitution of undefined residues by alanine, in the regulatory response domain (A) and in the Sigma Factor 54 domain (B) for each sampling point in the cities.

**A**

B

**Supplementary STable 2:** Frequency and distribution of ‘X’ residues in the analyzed sequences.

| **Sample** | **Total residues** | **Number of 'X'** | **Percentage of 'X** | **Average spacing between 'X'** |
| --- | --- | --- | --- | --- |
| Bragança | 478 | 15 | 3.14% | 31.71 |
| BP1 | 148 | 109 | 73.65% | 1.26 |
| BP2 | 478 | 11 | 2.30% | 44.40 |
| BP3 | 394 | 68 | 17.26% | 4.63 |
| BP4 | 479 | 104 | 21.71% | 4.64 |
| BP5 | 467 | 199 | 42.61% | 2.35 |
| Paragominas | 478 | 13 | 2.72% | 22.50 |
| PP1 | 126 | 93 | 73.81% | 1.30 |
| PP2 | 478 | 8 | 1.67% | 60.57 |
| PP3 | 478 | 29 | 6.07% | 10.86 |
| PP4 | 479 | 102 | 21.29% | 2.65 |
| PP5 | 62 | 59 | 95.16% | 1.02 |
